# Roux-en-Y gastric bypass surgery in Zucker rats induces bacterial and systemic metabolic changes independent of caloric restriction-induced weight loss

**DOI:** 10.1080/19490976.2021.1875108

**Published:** 2021-02-04

**Authors:** Florian Seyfried, Jutarop Phetcharaburanin, Maria Glymenaki, Arno Nordbeck, Mohammed Hankir, Jeremy K Nicholson, Elaine Holmes, Julian R. Marchesi, Jia V. Li

**Affiliations:** a Department of General, Visceral, Transplant, Vascular, and Pediatric Surgery, University Hospital Wuerzburg, Wuerzburg, Germany; b Department of Metabolism Digestion and Reproduction, Faculty of Medicine, Imperial College London London, UK; c Department of Biochemistry, Faculty of Medicine, Khon Kaen University, Khon Kaen, Thailand; d Division of Organisms and Environment, School of Biosciences, Institute of Health Futures, Murdoch University, Perth, Western Australia, Australia; e School of Biosciences, Cardiff University, Cardiff, UK

**Keywords:** Bariatric surgery/bile acids/metabolism/microbiota/microbiome

## Abstract

Mechanisms of Roux-en-Y gastric bypass (RYGB) surgery are not fully understood. This study aimed to investigate weight loss-independent bacterial and metabolic changes, as well as the absorption of bacterial metabolites and bile acids through the hepatic portal system following RYGB surgery. Three groups of obese Zucker (*fa/fa*) rats were included: RYGB (n = 11), sham surgery and body weight matched with RYGB (Sham-BWM, n = 5), and sham surgery fed *ad libitum* (Sham-obese, n = 5). Urine and feces were collected at multiple time points, with portal vein and peripheral blood obtained at the end of the study. Metabolic phenotyping approaches and 16S rRNA gene sequencing were used to determine the biochemical and bacterial composition of the samples, respectively. RYGB surgery-induced distinct metabolic and bacterial disturbances, which were independent of weight loss through caloric restriction. RYGB resulted in lower absorption of phenylalanine and choline, and higher urinary concentrations of host-bacterial co-metabolites (e.g., phenylacetylglycine, indoxyl sulfate), together with higher fecal trimethylamine, suggesting enhanced bacterial aromatic amino acid and choline metabolism. Short chain fatty acids (SCFAs) were lower in feces and portal vein blood from RYGB group compared to Sham-BWM, accompanied with lower abundances of *Lactobacillaceae*, and *Ruminococcaceae* known to contain SCFA producers, indicating reduced bacterial fiber fermentation. Fecal γ-amino butyric acid (GABA) was found in higher concentrations in RYGB than that in Sham groups and could play a role in the metabolic benefits associated with RYGB surgery. While no significant difference in urinary BA excretion, RYGB lowered both portal vein and circulating BA compared to Sham groups. These findings provide a valuable resource for how dynamic, multi-systems changes impact on overall metabolic health, and may provide potential therapeutic targets for developing downstream non-surgical treatment for metabolic disease.

## Introduction

The incidence of obesity continues to grow at an unprecedented rate posing a global health crisis.^1^ Bariatric surgery is currently the only effective treatment for morbid obesity, resulting in rapid and substantial weight loss that is sustained long-term.^2^ Roux-en-Y Gastric Bypass (RYGB) is amongst the most commonly performed bariatric procedures and has been shown to increase circulating anorexigenic and glucoregulatory gut hormone levels (e.g., glucagon-like peptide-1 (GLP-1) and peptide YY (PYY)), as well as to induce profound systemic metabolic and the gut microbial changes.^3,4^ An increase in the relative bacterial abundance of Gammaproteobacteria and Verrucomicrobia in feces has consistently been reported in both human and animal models post RYGB.^4–6^ These gut bacterial changes in the RYGB-operated patients are persistent for up to 9 years post-operatively, and are considered to regulate host metabolism and fat mass.^7^ Furthermore, metabolic phenotyping studies have revealed profound alterations in urinary host-microbial co-metabolites, such as phenylacetylglycine, phenylacetylglutamine, 4-cresyl sulfate, and trimethylamine *N*-oxide (TMAO), suggesting that RYGB induces both compositional and functional changes of the gut microbiota.^4,6^ However, there remains a lack of understanding concerning weight loss-independent systemic metabolic changes over time. In addition, emerging evidence indicates that bile acids (BAs) play a key role in the mechanisms of bariatric surgery.^8,9^ BAs are cholesterol-derived molecules and aid in the absorption of dietary fat. BAs, as ligands for the nuclear farnesoid X receptor (FXR), control their own synthesis and hepatic lipid metabolism.^10^ BAs also promote GLP-1 secretion from enteroendocrine cells^11^ and influence systemic metabolism by driving energy expenditure through activation of G-protein coupled receptor 5 (GPR5).^12^ BAs also regulate glucose metabolism by increasing insulin sensitivity and suppressing gluconeogenesis.^10^ Improved insulin sensitivity after RYGB has been linked to increased circulating secondary BAs, particularly glycodeoxycholic acid.^13^ However, it is unclear how bariatric surgery influences the absorption of bile acids and bacterial metabolites. The portal vein is the point of exit for a multitude of host-microbe signaling molecules emanating from the gastrointestinal tract, but little is known about how the portal vein blood metabolome is affected by RYGB. Therefore, to address these issues, we studied fecal bacterial composition and a collection of biofluids from obese Zucker rats at multiple time points, as well as investigated the absorption of bile acids and bacterial metabolites in the portal vein blood following RYGB surgery.

## Results

### The effect of RYGB and caloric restriction on food intake, body weight and oral glucose tolerance

**Figure 1.**
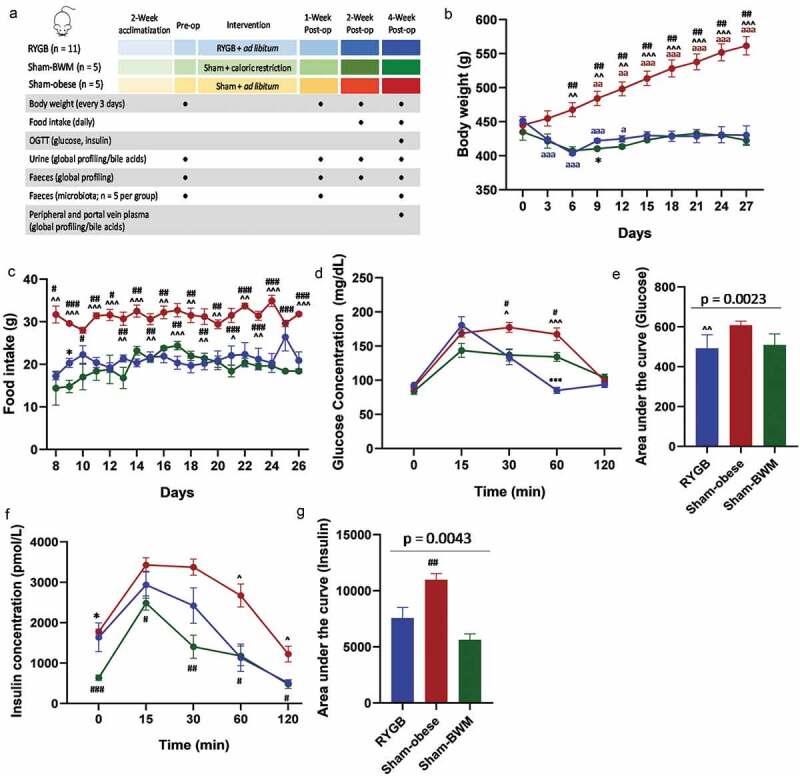
Impact of the Roux-en-Y gastric bypass surgery on phenotypic changes of Zucker obese rats

Twenty-one obese Zucker (*fa/fa*) rats were divided into three groups: RYGB (n = 11), sham surgery and body weight matched with RYGB (Sham-BWM, n = 5), and sham surgery with food *ad libitum* (Sham-obese, n = 5) (Figure 1a). Urine and feces were collected at pre-surgery, 1, 2, and 4 weeks post-surgery. Plasma samples including both portal vein and peripheral blood were collected at week 4.

Experimental design, sample collection and analyses (a); animal body weight (b) and food intake (c) throughout the experimental period; following an oral glucose tolerance test, plasma concentrations of glucose (d) and insulin (f), and the areas under the curves (AUCs) for glucose and insulin (e, g). Data are shown as mean±SEM and statistical differences were determined by two-way ANOVA with Tukey’s multiple comparisons test (b, c, d & f), whereas Kruskal-Wallis test was used to determine the significance level of the AUCs among the RYGB (blue), sham-obese (red) and sham-BWM (green) groups. Dunn’s multiple comparisons test was used for pairwise comparisons of the AUCs between any of the two groups. * indicates statistically significant differences between RYGB and Sham-BWM (* *p* < .05; ** *p* < .01; *** *p* < .001). ^ indicates statistically significant differences between RYGB and Sham-obese (^^^*p* < .05; ^^^^*p* < .01; ^^^^^*p* < .001). # indicates statistically significant differences between Sham-BWM and Sham-obese (^#^*p* < .05; ^##^*p* < .01; ^###^*p* < .001). ^a^ indicates statistically significant differences between post surgical time points and pre-surgery of the same intervention group (^a^*p* < .05; ^aa^*p* < .01; ^aaa^*p* < .001).

Within 6 days of intervention, mean body weights of RYGB and Sham-BWM rats were significantly reduced compared to pre-intervention, reaching a plateau (~430 g) over the remaining period of the study (Figure 1b). Mean body weight of Sham-obese rats was significantly higher than that of either RYGB or Sham-BWM groups from 6 days post-surgery onwards (Figure 1b). While Sham-obese rats consumed a significantly higher amount of food than RYGB and Sham-BWM groups, the food intake of RYGB and Sham-BWM were similar despite the different weight loss strategies (Figure 1c). An oral glucose tolerance test (OGTT) showed significantly lower concentrations of plasma glucose in the RYGB group compared with Sham-obese (*p* = .0005) or Sham-BWM (*p* = .0008) groups at 60 mins following an oral dose of glucose, while sham-BWM exhibited significantly lower levels than the Sham-obese group (*p* = .048) (Figure 1d). The area under the curve (AUC) of the glucose concentrations over 120 min of OGTT was significantly lower in RYGB-operated rats than in Sham-obese rats (Figure 1e). Consistent with their insulin resistance, Sham-obese rats had the highest insulin levels among the three groups during the OGTT (Figures 1e and 1f).

## RYGB alters fecal bacterial composition

**Figure 2.**
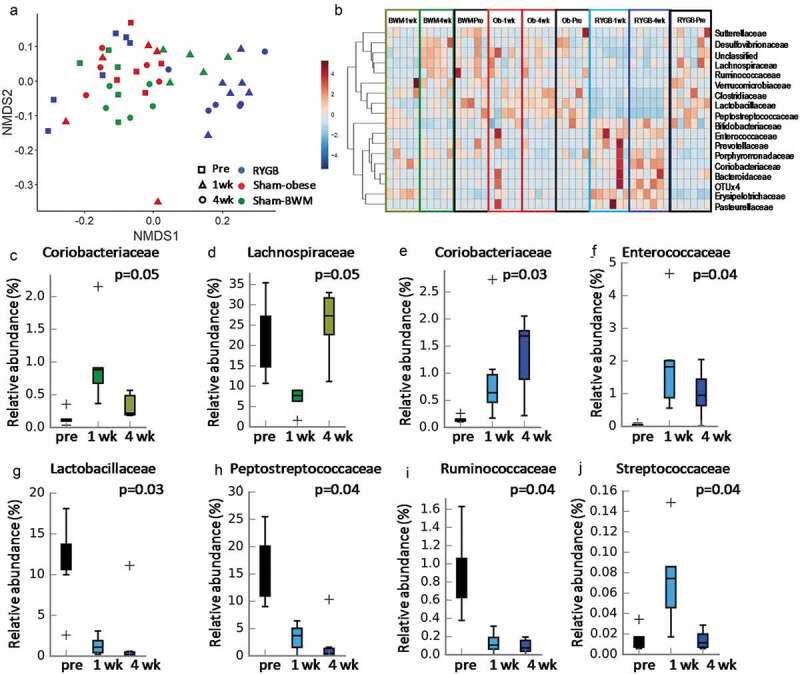
Impact of the Roux-en-Y gastric bypass surgery on fecal microbial profiles of Zucker obese rats

There was no significant difference in Shannon diversity index, species richness, or Chao1 between RYGB and Sham-obese or Sham-BWM, except for a significantly higher Shannon diversity index in Sham-BWM compared to the other two groups at week 4 (Fig S1). These alpha diversity measurements were not significantly different between time points within each experimental group. While all the animals had a similar starting microbiota, RYGB rats experienced deviation at a larger scale over time, whereas Sham-obese remained tightly clustered and Sham-BWM showed a temporary diversification at week 1 before retroceding to the same multivariate space occupied by pre-surgery samples (Figure 2a, Fig S2A). Bacterial families such as *Lactobacillaceae* and *Peptostreptococcaceae* were reduced post-RYGB surgery but not in the Sham groups (Figure 2b). In the Sham-BWM group, the relative abundance of *Coriobacteriaceae* and *Lachnospiraceae* were significantly changed at week 1 and shifted back to pre-intervention level (Figure 2c-2d). In contrast, *Coriobacteriaceae* and *Enterococcaceae* were consistently increased, whereas *Lactobacillaceae, Peptostreptococcaceae, Ruminococcaceae* were reduced post-RYGB (Figure 2e-2i). *Streptococcaceae* increased at 1-week post-RYGB and shifted back to pre-surgery level by week 4 (Figure 2j).

A NMDS plot (a) and a heatmap (b) of fecal family-level taxonomic profiles from RYGB, sham-obese and sham-BWM groups at pre-surgery (Pre), and 1- (1 wk) and 4-weeks (4 wk) post-surgery. Clustering analysis was based on the Pearson correlation coefficient as the distance measure between data points and the average linkage as clustering algorithm. Box plots show significantly changed bacterial families including *Coriobacteriaceae* (c) and *Lachnospiraceae* (d) in sham-BWM group and *Coriobacteriaceae* (e), *Enterococcaceae* (f), *Lactobacillaceae* (g), *Peptostreptococcaceae* (h), *Ruminocoaccaceae* (i), and *Streptococcaceae* (j) in RYGB group across three measured time points. Kruskal-Wallis H-test and Tukey-Kramer post-hoc test were used and Benjamini-Hochberg FDR was used for multiple test correction.

We did not observe any significant changes among the groups at pre-surgery, nor between Sham-obese and Sham-BWM at any time point. However, the RYGB group showed significantly lower abundances of *Peptostreptococcaceae* at week 1 and unclassified Clostridiales, *Ruminococcaceae* and *Lachnospiraceae* at week 4, while there was a higher abundance of *Enterococcaceae* at week 1 compared with Sham-BWM (Fig S2B). When comparing Sham-obese to RYGB, higher levels of *Erysipelotrichaceae* and lower levels of *Lactobacillaceae* were observed at both week 1 and 4 in RYGB animals. Furthermore, *Peptostreptococcaceae, Clostridiaceae*, and unclassified Clostridiales were found to be underrepresented in RYGB versus Sham-obese, whereas *Coriobacteriaceae* were overrepresented.

## RYGB-induced weight loss-independent global metabolic changes in feces over time

**Figure 3.**
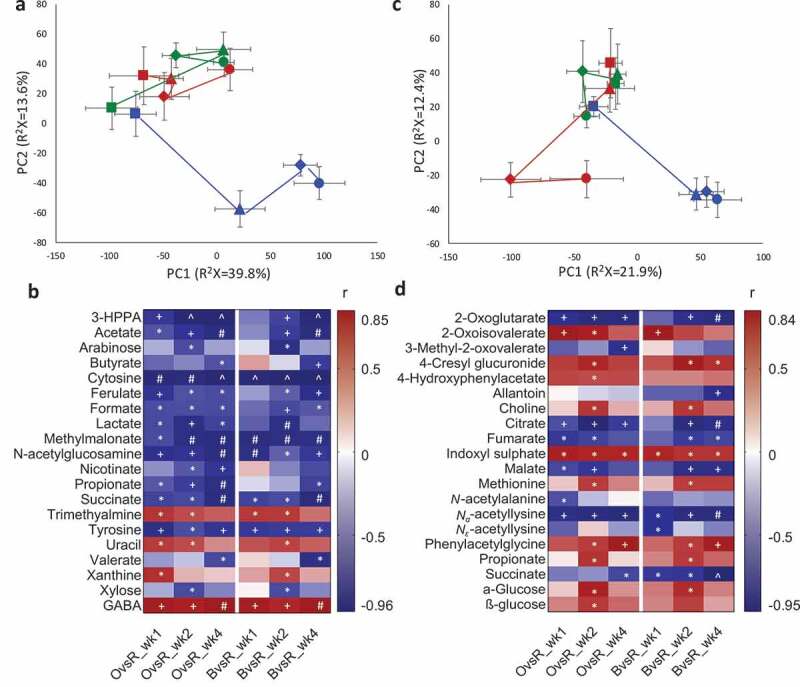
Impact of the Roux-en-Y gastric bypass surgery on fecal and urinary metabolic profiles of Zucker obese rats

To investigate the impact of RYGB on fecal metabolite composition, we used ^1^H NMR spectroscopy to analyze fecal water extracts from RYGB, Sham-BWM, and Sham-obese groups at pre- and 1-, 2- and 4-week post-surgery and a PCA scores plot showed a sustained impact of RYGB surgery at 4 weeks compared to transient effects of the sham-operated animals (Fig S3A). A more detailed temporal analysis using PCA trajectory plots, calculated from the PCA scores plots, showed that prior to RYGB or sham surgery, all animals had similar baseline fecal metabolomes, which started to deviate as early as 1-week post intervention (Figure 3a). The RYGB group showed a greater metabolic shift from pre-surgery to post-surgery time points along both the first and second principal components (PC1 and PC2). In contrast, the Sham-BWM and Sham-obese groups deviated along PC1 (Figure 3a). PCA and O-PLS-DA analyses were subsequently carried out to specifically investigate (1) caloric restriction-induced metabolic changes by comparing the Sham-BWM with the Sham-obese group; (2) RYGB-induced metabolic changes by comparing the RYGB with the Sham-obese group; and (3) RYGB-induced body weight-independent metabolic changes by comparing the RYGB with the Sham-BWM group. There were no significant metabolic differences in fecal water among the three groups of animals at pre-surgery. Given that caloric restriction-induced significant body weight loss, we did not observe any significant metabolic changes in feces between Sham-BWM and Sham-obese groups (Table S1). However, significant metabolic differences between RYGB and either of the Sham groups were observed at post-surgery time points (Figs S4-S5). A heatmap (Figure 3b) demonstrates significant metabolic differences between RYGB and Sham-obese or Sham-BWM. RYGB-operated rats showed higher fecal levels of γ-amino butyric acid (GABA), and lower levels of succinate, *N*-acetylglucosamine, cytosine, methylmalonate and tyrosine over the 4-week postoperative period compared with Sham-BWM or Sham-obese. In contrast, short chain fatty acids (SCFAs), such as formate and propionate, lactate and 3-hydroxyphenylpropionate (3-HPPA) were lower throughout the post-surgery period in the RYGB than in the Sham-obese group, whereas these metabolite changes were only observed at one or two time points in the comparison between Sham-BWM and RYGB groups. While trimethylamine was higher in RYGB compared to Sham groups at week 1 and 2 post-op, SCFAs including acetate, valerate and butyrate were lower at later time points. Notably, a smaller number of changed metabolites between RYGB and Sham-BWM at week 1 was observed in contrast to the other comparisons, suggesting that caloric restriction induced an initial but temporary metabolic disturbance. These observations suggested that body weight reduction through caloric restriction and RYGB manifest different metabolic patterns.

PCA trajectory scores plots of fecal water (a) and urine (c) based on ^1^H NMR spectral data obtained from RYGB (blue), sham-BWM (green) and sham-obese (red) Zucker rats at pre-surgery (square), 1- (triangle), 2- (diamond) and 4-week (circle) post-surgery. Each dot represents the average scores of the first principal component (PC1) against the second principal component (PC2) from each group. Error bars are expressed by the standard error of the mean of PC1 and PC2 at each time point. Heatmaps of significantly changed metabolites between RYGB and Sham-obese (OvsR) or Sham-BWM (BvsR) in feces (b) and urine (d) at each time points (1, 2 and 4 weeks). Correlation coefficient (r) shown in the heatmaps were obtained from OPLS-DA models. Positive (red) or negative (blue) r values indicate higher or lower concentrations of the metabolites in RYGB compared with either of the sham groups. *, +, # and ^ indicate significant levels of metabolite differences between pairwise groups at each time point at *p* < .05, *p* < .01, *p* < .001 and *p* < .0001, respectively using two-tailed heteroscedastic t-test with Benjamini-Hochberg correction. Keys: 3-HPPA, 3-Hydroxyphenylpropionate; GABA, γ-amino-butyric acid.

## RYGB induces weight loss-independent global metabolic changes in urine over time

Urinary profiles of the Sham-BWM group presented a tight clustering of all time points, while Sham-obese and RYGB groups shifted dramatically, forming three distinctive clusters representing each experimental group (Figure 3c and S3B). There was no significant difference in the urinary profiles between Sham-BWM and Sham-obese at any time points (Table S1). In contrast, metabolic differentiation RYGB and Sham-obese or Sham-BWM was observed over all post-surgical time points (Figs S6-S7). The concentrations of TCA cycle intermediates (e.g., citrate, 2-oxoglutarate, succinate, fumarate and malate) were lower in RYGB compared with Sham groups, whereas the host-microbial co-metabolites including indoxyl sulfate (IS), 4-hydroxyphenylacetate, 4-cresyl glucuronide and phenylacetylglycine (PAG) were higher (Figure 3d). Urinary levels of 2-oxoisovalerate, the breakdown products of branched chain amino acid (BCAA), valine, were found to be higher in RYGB than in Sham animals. However, 3-methyl-2-oxovalerate, a breakdown product of isoleucine, was lower in RYGB compared to Sham-obese. Furthermore, *N_ε_*-acetyllysine, *N*-acetylalanine and *N_α_*-acetyllysine were found to be lower in RYGB.

## RYGB affects biochemical composition of peripheral and portal vein blood plasma

**Figure 4.**
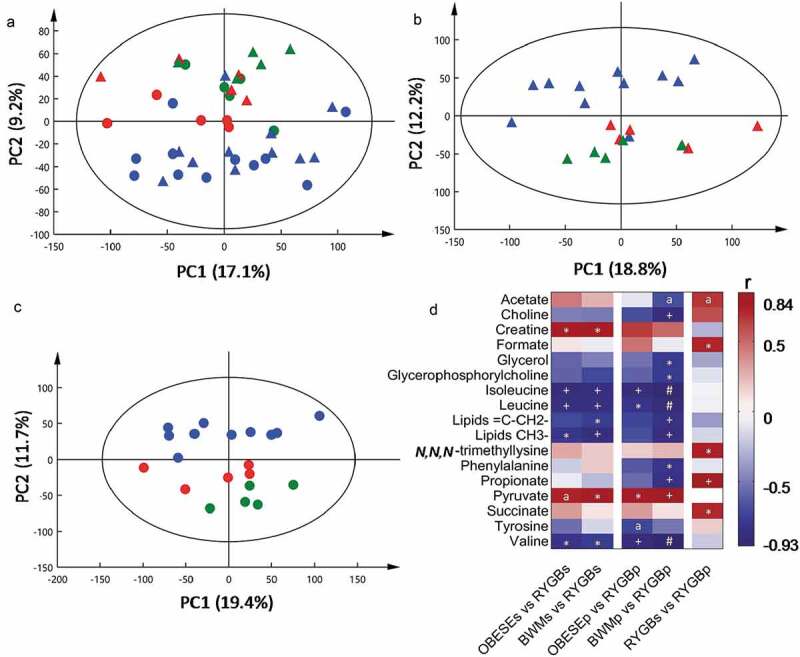
Impact of the Roux-en-Y gastric bypass surgery on portal vein and peripheral plasma metabolic profiles of Zucker obese rats

*Portal vein vs. peripheral plasma*: The PCA scores plot of the peripheral and portal vein plasma from all three groups showed a clear clustering based on experimental groups (Figure 4a-4c). RYGB animals were separated from the Sham animals along PC2, whereas Sham-BWM and Sham-obese appeared to group along PC1. While neither Sham-BWM nor Sham-obese demonstrated differences between portal vein and peripheral plasma (Table S1), the RYGB group showed higher levels of *N,N,N-*trimethyllysine, propionate, formate, and succinate in the portal vein than in peripheral plasma (Figure 4d). Acetate levels were also high but did not reach a statistical significance post Benjamini-Hochberg (BH) correction (*p* = .06).

PCA scores plots (a, b, c) of ^1^H NMR spectral data obtained from peripheral (dot) and portal vein (triangle) samples from RYGB (blue), sham-BWM (green) and sham-obese (red) Zucker rats at 4-week post-operation. A heatmap (d) of significantly changed metabolites between RYGB and Sham-obese or Sham-BWM in systemic (s) and portal vein blood plasma (p). Correlation coefficient (r) shown in the heatmap were obtained from OPLS-DA models. Positive (red) or negative (blue) correlation coefficient values indicate higher or lower concentrations of the metabolites in RYGB or RYGB portal vein compared with either of the sham groups or RYGB peripheral plasma. *, + and # indicate significant levels of metabolite differences between the groups at *p* < .05, *p* < .01 and *p* < .001, respectively, using two-tailed heteroscedastic t-test with Benjamini-Hochberg correction. ^a^ Metabolites with a *p* = .06 post BH correction.

*RYGB vs Sham groups*: Regardless of peripheral or portal vein plasma, the two Sham groups exhibited no significant differences (Table S1). However, RYGB rats showed significantly lower levels of BCAA (i.e. leucine, isoleucine and valine) and higher levels of pyruvate in contrast to Sham groups (Figure 4d). Furthermore, portal vein blood levels of choline, phenylalanine, glycerol, glycerophosphorylcholine and propionate were lower in RYGB compared with the Sham-BWM. Portal vein blood levels of tyrosine was lower in RYGB compared to Sham-obese but did not reach statistical significance post BH correction (*p* = .06).

## RYGB significantly alters bile acid composition in portal vein and peripheral plasma but not in urine

BAs released during digestion are re-absorbed back to the liver from the distal ileum through the enterohepatic circulation. Therefore, BA profiles of the portal vein blood reflect the ileal re-absorption, whereas that of peripheral blood and urine represent the circulating and excreted BAs, respectively. We did not observe significant BA differences between RYGB and Sham groups after applying multiple testing corrections.

**Figure 5.**
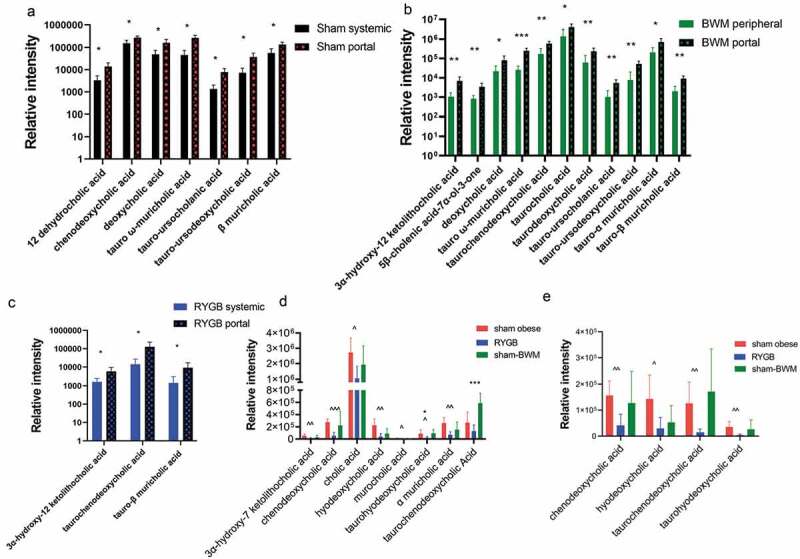
Impact of the Roux-en-Y gastric bypass surgery on portal vein and peripheral plasma bile acids of Zucker obese rats

*Portal vein vs. Peripheral plasma*: We identified 32 bile acids in plasma samples using a UPLC-MS-based bile acid profiling method (Table S2). A total of 7, 11, and 3 bile acids were significantly higher in the portal vein compared to peripheral plasma in Sham-obese, Sham-BWM and RYGB groups, respectively (Figure 5a-5c). Caloric restriction had a more profound impact on bile acids than RYGB did. RYGB disturbed bile acid circulation and metabolism, resulting in a more similar bile acid composition between the peripheral and portal vein plasma. 3α-hydroxy-12 ketolithocholic acid, taurochenodeoxycholic acid and tauro-β muricholic acid, were found to be higher in the portal vein compared to peripheral plasma in both RYGB and Sham-BWM, but not in the Sham-obese group. Furthermore, higher levels of deoxycholic acid, tauro-α muricholic acid, tauro-ursocholanic acid, and tauro-ursodeoxycholic acid were observed in portal vein compared to systemic levels in both Sham groups.

Bile acids that significantly differed between portal vein and peripheral plasma in sham-obese (a), sham-BWM (b) and RYGB (c) groups, and bile acids that significantly differed between RYGB and sham-obese or sham-BWM (*p* < .05 by multiple t-test with FDR correction) in portal vein (d) and in peripheral plasma (e). Bars are presented by mean±SD. * indicates statistically significant differences between peripheral and portal vein plasma or between RYGB and Sham-BWM (* *p* < .05; ** *p* < .01; *** *p* < .001). ^ indicates statistically significant differences between RYGB and Sham-obese (^^^*p* < .05; ^^^^*p* < .01; ^^^^^*p* < .001).

*RYGB vs. Sham groups*: The levels of bile acids in the RYGB group were lower than those in the Sham groups. The RYGB group showed lower levels of primary bile acids (e.g., cholic acid, chenodeoxycholic acid and α-muricholic acid) and secondary bile acids (e.g., hyodeoxycholic acid and 3α-hydroxy-7 ketolithocholic acid) compared with Sham-obese in the portal vein plasma (Figure 5d). Taurohyodeoxycholic acid and taurochenodeoxycholic acid were significantly lower in RYGB compared to Sham-BWM (Figure 5d). Unlike in portal vein plasma, levels of only 4 BAs were altered in peripheral plasma between RYGB and Sham-obese. These include taurine-conjugated BAs, such as taurochenodeoxycholic acid and taurohyodeoxycholic acid, as well as the unconjugated chenodeoxycholic acid, and hyodeoxycholic acid (Figure 5e). While similar trends of these changes were observed between RYGB and Sham-BWM groups, no statistical significance was achieved.

## Discussion

**Figure 6.**
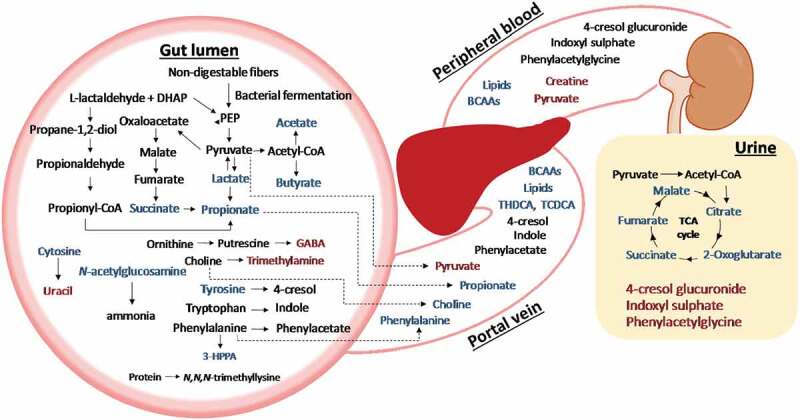
Schematic illustration of systemic changes observed in biofluids from RYGB-operated obese rats compared to Sham-BWM

RYGB surgery induced a range of metabolic changes over a 4-week period post-surgery independent of weight loss through caloric restriction (Figure 6). The levels of bacterial metabolites, such as urinary indoxyl sulfate, and fecal succinate, tyrosine and GABA, changed as early as the first post-surgical time point (i.e., 1-week post-op) and these changes differentiating the RYGB from the Sham groups were consistent across the 4-week study period. However, other host-bacterial co-metabolites including PAG and 4-cresyl glucuronide were found to be significantly different between the RYGB and the sham groups only at later time points (i.e., 2- and 4-week post-op). SCFA production through bacterial fermentation of dietary fiber was significantly altered. Fecal levels of SCFAs such as formate, acetate, and propionate were consistently lower in RYGB group compared to Sham-obese at all post-surgical time points. However, in contrast to Sham-BWM, these changes were observed in RYGB group at later time points (i.e. 2 and 4 weeks), suggesting that caloric restriction may partially contribute to these reduced SCFAs. Caloric restriction has been reported to reduce total cecal SCFA levels^14^ and microbial butyrogenesis and acetogenesis in rats.^15^ This observation is consistent with the reduced relative cecal acetate in RYGB-operated mice compared to both Sham-obese and Sham-BWM mice previously reported, although butyrate was reported to be unchanged and propionate increased.^16^ Our observations suggested a reduced bacterial activity of fiber fermentation post-RYGB, which is likely due to reduced bioavailability of fiber in the colon and/or shifted bacterial composition. However, in this study, Sham-BWM and RYGB groups had similar food intake and therefore, the reduced SCFA levels in feces are likely to due to the bacterial compositional changes. This fact agrees with our observation that bacterial families, *Ruminococcaceae* and *Lactobacillaceae*, were found to be significantly reduced post RYGB, together with a trend towards lower abundance of *Lachnospiraceae*, which was not statistically significant. Bacteria from these families have been reported to produce SCFAs, for example, *Faecalibacterium prausnitzii* and *Subdoligranulum variabile* from *Ruminococcaceae, Roseburia inulinivorans*, and *Eubacterium rectale* from *Lachnospiraceae*, and *Lactobacillus reuteri* from *Lactobacillaceae*.^17^ Butyrate is the primary energy source of colonocytes^18^ and its decreased availability in feces may indicate an increase in other energy generation pathways, such as glycolysis or β-oxidation to compensate for the energy demands. A study on antibiotic-induced microbiome depletion demonstrated a shift in energy metabolism from SCFAs to glycolysis in enterocytes, affecting glucose homeostasis.^18^ Butyrate is believed to be a protective metabolite in the gut, implicates in the maintenance of gut integrity and has an anti-inflammatory role;^19^ therefore, its decrease may result in a reduction of these beneficial effects post RYGB. Moreover, a significantly higher levels of propionate in the portal vein blood was observed in Sham-BWM but not in Sham-obese compared to RYGB rats. Notably, fecal propionate was higher in Sham-obese across 4 weeks but not in Sham-BWM compared to RYGB. These findings together suggested that caloric restriction could result in a higher uptake of propionate via portal vein blood. It has been shown that 6-week caloric restriction in rats promoted the expression of microbial enzymes involved in propionogenesis.^15^ Propionate has been shown to reduce hepatic and serum fatty acid levels, the elevated levels of which can cause inflammation.^20^ Propionate may indirectly contribute to improved insulin sensitivity since both elevated-free fatty acids and inflammation can result in insulin resistance.^20^ This observation is consistent with our phenotypic data from the Sham-BWM animals, exhibiting similar insulin and glucose concentrations as compared to RYGB group following an oral glucose tolerance test. Propionate can also impact on food intake through GPR signaling, inducing the release of satiety hormones such as GLP-1 and PYY,^21^ as well as controlling energy expenditure by GPR41 signaling.^22^ Our data indicated that caloric restriction increased absorption of propionate, which in turn could potentially impact on food intake, forming a positive feedback loop.

Red indicates higher relative concentration, whereas blue indicates lower relative concentration of metabolites in RYGB compared with Sham-BWM. Key: GABA, γ-amino butyric acid; PEP, phosphoenolpyruvate; TCA cycle, tricarboxylic acid cycle; TCDCA, taurochenodeoxycholic aicd; THDCA, taurohyodeoxycholic acid.

Levels of microbially-derived neuroactive intermediate, GABA, were higher in RYGB-operated animals compared to Sham groups, which is in agreement with previous studies on rodent models of RYGB.^6^ Bacterial genera, such as *Enterococcus, Bacteroides*, Escherichia, and *Klebsiella*, have been reported to be involved in GABA biosynthesis.^23^ We observed that *Enterococcus* spp. was present in higher abundance post-RYGB. In addition, GABA has been reported to regulate gut motility^24^ and may affect food transition in the gut post-RYGB. GABA also stimulates release of GLP-1, which regulates glucose homeostasis by improving insulin secretion, from enterocytes through ionotropic GABA_A_ and GABA_C_ receptors.^25^ The increase in GLP-1, which is consistently observed in patients and animals post RYGB surgery,^3^ can in turn promote GABA production from pancreatic β-cells, creating a loop maintaining fecal and plasma GABA levels.^6^ We observed that RYGB animals exhibited improved insulin levels and glucose tolerance compared to Sham-obese, suggesting that microbially-derived GABA may play a role in surgery-induced metabolic benefits.

Aromatic amino acids (e.g., phenylalanine and tyrosine) and their metabolites were altered by RYGB surgery in obese Zucker rats. We found lower fecal levels of tyrosine and 3-HPPA, together with higher levels of urinary 4-cresyl glucuronide, 4-hydroxyphenylacetate, indoxyl sulfate, and PAG in RYGB-operated animals compared to Sham groups. In portal vein plasma, the uptake of phenylalanine and tyrosine was reduced in RYGB compared to Sham groups. These observations collectively led us to conclude that the absorption of aromatic amino acids was reduced post-RYGB and their bioavailability in the colon is likely to increase, allowing the gut bacteria to produce *p*-cresol and phenylacetate from tyrosine and phenylalanine, respectively. Consistent with the findings in a recent study by Haange *et al*.^5^ the concentrations of aromatic amino acids were reported to be lower in the cecum and colon content following RYGB, whereas the amines such as dopamine and histamine formed by Proteobacteria via decarboxylation of the corresponding precursor amino acids were higher. *P*-cresol and phenylacetate were subsequently taken into the liver for conjugation with glucuronide or glycine to form 4-cresyl glucuronide and PAG, respectively. 3-HPPA is a bacterial metabolite formed from phenylalanine. Our finding suggested that RYGB could facilitate greater conversion of phenylalanine to phenylacetate than to 3-HPPA. High indoxyl sulfate levels may lead to adverse effects on kidney and heart function since it is a uremic toxin associated with atherosclerosis.^26^ However, this metabolic observation conflicts with the clinical observation of improved heart function post-bariatric surgery. Increased aromatic amino acid metabolism by the gut microbiota may indicate potential harmful effects of RYGB in the long term since a meta-analysis on functional microbial signatures in colorectal cancer displayed an enrichment in amino acid degradation pathways.^27^ In addition, bacterial choline metabolism was enhanced by RYGB surgery, reflected by higher fecal trimethylamine, a bacterial product of choline and lower absorption of choline through the hepatic portal vein in the RYGB compared to Sham groups. Metagenomic analysis of colorectal cancer datasets further showed choline trimethylamine-lyase gene was overabundant in colorectal cancer patients.^28^ Moreover, there is evidence that bariatric surgery could increase the risk of colon cancer development in the long-term.^29,30^

The fecal bacterial composition was temporarily affected by caloric restriction with a higher abundance of *Coriobacteriaceae* and a lower abundance of *Lachnospiraceae* at week 1 compared to pre-intervention and these changes normalized by week 4. Low abundances of *Lachnospiraceae* were consistent with the previously reported taxa that are associated with caloric restriction.^31^ While caloric restriction has been reported to induce changes in levels of amino acids, ketone bodies, bile acids, and host-microbial co-metabolites,^31^ we did not observe significant differences in the metabolic profiles between Sham-obese and Sham-BWM using multivariate statistical analysis methods. This result could be owing to a relatively short period of caloric restriction (4 weeks) and/or a small group size of the sham groups. The abundance of *Coriobacteriaceae* was found to be significantly increased following RYGB, which corroborates a recent report on diabetic rats post-RYGB.^32^ *Coriobacteriaceae* statistically and negatively correlated with hepatic glucose and glycogen in germ-free mice exposed to a conventional environment, suggesting a potential impact of *Coriobacteriaceae* on glucose metabolism. In the current study, given that Sham-BWM and RYGB rats exhibited a similar body weight loss, the relative abundances of fecal bacterial families such as *Peptostreptococcaceae, Ruminococcaceae*, and *Lachnospiraceae* were found to be higher in Sham-BWM compared to RYGB, suggesting that caloric restriction- and RYGB-induced weight loss could be associated with different bacteria. This is supported by a pilot study comparing the fecal bacterial composition of patients who underwent medical weight loss (MWL), adjustable gastric banding (AGB), or RYGB.^33^ With a similar weight loss and glycemic improvement, the study showed that RYGB induced the most number of bacterial species shifts compared to the other two groups and alpha-diversity was significantly lower in AGB compared to RYGB and MWL.^33^ Consistently, in a diabetic rat model, RYGB and sleeve gastrectomy induced similar glucose improvement but produced distinct fecal bacterial signatures.^34^ Another study based on fecal microbiota transplantation showed that mice receiving the fecal microbiota from patients with poor weight loss post-RYGB exhibited a greater weight gain compared to those who were transplanted with the microbiota from the successful responders.^35^ While no statistical differences in the fecal microbial composition were found between poor and successful weight loss responders, *Barnesiella* was observed to be associated with weight gain in the recipient mice.^35^ Furthermore, de Jonge *et al*. showed that nonsurgical duodenal–jejunal bypass liner, which excludes the proximal 60 cm small intestine from food, induced an increased fecal abundance of Proteobacteria, but that this shift reverted to baseline levels after removal of the liner, while the weight loss persisted.^36^ These observations suggested that these fecal microbial shifts are likely to be independent of weight loss.

Bile acids are believed to play a key role in achieving metabolic benefits of RYGB surgery.^37^ However, while the majority of studies in animal models and patients have shown a surge in plasma BA levels postoperatively,^38,39^ others reported no change^40^ or even a reduction.^41^ Similarly, fecal bile acids have been shown to markedly decrease in patients and rats after RYGB,^6,42,43^ raising the question of the role of luminal bile acids in regulating metabolic function. In this study, we showed that BA levels in portal vein were higher compared to peripheral blood in all three groups of animals, with the highest number of disturbed BAs in Sham-BWM, suggesting caloric restriction has a greater impact on BA composition. Three BAs, namely, 3α-hydroxy-12-ketolithocholic acid, taurochenodeoxycholic acid, and tauro-β-muricholic acid were found to be higher in portal vein blood in both RYGB and Sham-BWM groups compared to their systemic levels, which could be due to reduced food intake following the surgery. Moreover, primary BAs such as cholic acid and chenodeoxycholic acid, and secondary BAs, mainly taurine-conjugated BAs (e.g., taurohyodeoxycholic acid and taurochenodeoxycholic acid) were present at lower levels in RYGB compared to Sham-operated groups, which is consistent with the observations in a mini-pig model post-RYGB.^44^ Notably, ileal conjugated bile acids are also lower in RYGB-operated compared to Sham-obese rats,^45^ suggesting that their increased deconjugation by ileal microbiota account for their decreased reabsorption into the enterohepatic circulation. Simonen and colleagues^39^ showed that despite total serum BA levels increasing post RYGB, levels of conjugated BAs, specifically taurine-conjugated BAs, decreased and this change was associated with increased lipid oxidation at the expense of glucose oxidation. This data corroborates our observations of reduced plasma lipids and glycerophosphorylcholine. FXR signaling, mainly activated by chenodeoxycholic acid, modulates BA synthesis, lipid metabolism, glucose homeostasis, and inflammation.^46^ Taurine-conjugated BAs are the most potent activators of TGR5 receptor.^46^ TGR5 signaling is implicated in energy balance, GLP-1 secretion, glucose metabolism and insulin sensitivity.^11,47^ However, the reduction of plasma BA levels observed in our study may suggest that the axis of BAs and TGR5/FXR signaling may not be the key mechanism, through which RYGB improves glucose tolerance and insulin sensitivity in Zucker obese rats, which is in agreement with a previous study carried out using TGR5-/- mice.^48^ A growing body of evidence suggests that temporal changes in BA levels could be unrelated to glycemic control early after gastric bypass.^49^ Furthermore, disruption of enterohepatic circulation using BA sequestrants^50^ or inhibitors of BA transporters^51^ resulting in reduced plasma BAs and increased fecal excretion nonetheless improved glucose metabolism.

An increase in TCA cycle activity post-RYGB was observed, as reflected by decreased urinary levels of TCA cycle intermediates and increased plasma levels of pyruvate, which are consistent with previous findings.^6,52^ Furthermore, it was noted that these intermediates apart from succinate were not significantly changed between Sham-BWM and RYGB until week 2 post-intervention, indicating that caloric restriction increased TCA cycle activity temporarily, whereas RYGB exerted a more sustained effect on TCA cycle. The high pyruvate level we observed in both portal vein and peripheral plasma after RYGB denotes augmented glycolysis and inhibition of gluconeogenesis. Another study on RYGB-operated rodents has reported increased glucose uptake and utilization in the Roux limb of the intestine.^53^ Therefore, stimulated glycolysis and resulting increased TCA cycle flux could contribute to increased energy expenditure post-RYGB.

Systemic metabolic profiles of obese Zucker rats collectively demonstrated that RYGB surgery reduced bacterial fermentation activities of fiber, and increased choline and host-microbial co-metabolisms, which are weight loss independent. For the first time, we studied the biochemical composition of both peripheral and portal vein plasma post-RYGB surgery and found reduced absorption of choline, aromatic amino acids and SCFAs, as a result of anatomic alteration of the intestine and bacterial compositional and functional changes. These findings provide a valuable resource for how dynamic, multi-systems changes impact on overall metabolic health, and may provide potential therapeutic targets for developing downstream non-surgical treatment for metabolic disease.

## Materials and methods

### Animals and experimental design

All experiments were approved by the Veterinary Office of the Government of Unterfranken, Germany (License 55.2–2531.01-72/12). Twenty-one male Zucker fa/fa rats at 6 weeks of age with a mean body weight of 327 ± 18 g were purchased from Charles River (France). Male rats were used to avoid confounders introduced by the estrous cycle since it has been shown that estradiol-treated ovariectomized rats exhibited a higher weight loss post-RYGB compared to non-treated control rats.^54^ Animals were individually housed under ambient humidity and temperature for 22°C in a 12 h light/dark cycle. Animals had free access to tap water and Purina 5008 Lab diet (Purina Mills, USA, 16.7% of calories from fat) unless stated otherwise. At 12 weeks of age, 11 rats were subjected to RYGB surgery and the other 10 were undergone sham surgery. After surgery, RYGB (n = 11) and sham-operated (n = 5, Sham-obese) rats were fed *ad libitum*. The other five sham-operated rats were calorie restricted and received the amount of calories necessary to achieve the same body weight as the RYGB group (Sham-BWM). The group size was determined based on previous studies.^55,56^ The study design is shown in Figure 1a. Food intake and body weight were measured daily. As regulation of glucose control and the secretion of the gastrointestinal peptide hormones are under circadian rhythm,^57^ we measured food intake every 2 hours in Sham-obese and RYGB rats. Based on these results, 33% of the daily food intake of Sham-obese rats was fed to Sham-BWM rats during the light phase, and 66% at the beginning of the dark phase. We performed all metabolic measurements at the beginning of the dark phase to avoid possible confounders related to the circadian rhythm.

## Surgery and perioperative care

Rats were food deprived for 6 h pre-operatively. Surgical anesthesia was induced and maintained with isoflurane/O_2_ mixture. Animals were placed on a heating pad during surgery. Prior to surgery animals were given 5 mg/kg carprofen subcutaneously. The abdomen was opened using a midline laparotomy and closed using continuous suturing. Sham surgery: The small bowel and gastro-esophageal junction were mobilized and a gastrostomy on the anterior wall of the stomach and a jejunostomy with subsequent closure were performed. Eleven rats underwent sham surgery and were included in the study. RYGB surgery: Surgery was performed according to a standardized protocol which has been shown to result in weight loss and its long-term maintenance. Briefly, the jejunum was transected 16 cm aboral to the pylorus to create the biliopancreatic limb. The stomach was divided 3 mm below the gastro-esophageal junction to create a small pouch. The stomach remnant was subsequently closed. The aboral jejunum was anastomosed end-to-side to the small pouch. At the level of the lower jejunum, a 7 mm side-to-side jejuno-jejunostomy between the biliopancreatic limb and the alimentary limb was performed creating a common channel of ~25 cm in length.

## Measurement of oral glucose tolerance, glucose and fasting insulin

An oral glucose tolerance test (OGTT) was performed at the beginning of the dark cycle in all animals 2 days prior to the biofluid sample collection at week 4 post-operation. In order to avoid oral gavage and therefore to reduce discomfort and pressure on the upper anastomosis, animals were trained to drink 10 ml/kg body weight of a 25% glucose solution within 10 min after an overnight fast on two occasions before the OGTT was performed. After an 8-h overnight fast, blood glucose was measured (Breeze 2® glucometer, Bayer, Zurich, Switzerland) in conscious rats at baseline, and 15, 30, 60, and 120 minutes after glucose ingestion. Blood was obtained from the tail vein by a small incision. A drop of blood was applied directly to a glucometer and 100 µl were collected at each time point in tubes containing EDTA and a dipeptidyl peptidase-4 inhibitor for insulin measurement. The plasma fraction was separated by centrifugation at 4°C, 8000 rpm and stored at −80°C. Total insulin was measured using the Ultrasensitive Rat Insulin ELISA (Merodia AB, Sweden 10–1251-10).

## Sample collection

Urine and feces were collected prior to the surgery (pre-op) and 1, 2, and 4 weeks post-surgery using metabolic cages (Techniplast, 3701M081) during the dark phase. Animals were provided with free access to water and diet. At the sacrifice time point, hepatic portal vein and peripheral blood were collected, and plasma samples were obtained from each animal.

## NMR spectroscopic analysis

Approximately 250 mg of homogenized fecal samples were mixed with 500 μL HPLC-grade water in 1.5 mL microtubes. The mixtures were sonicated for 3 × 10 minutes at 25°C and vortexed when each sonication cycle was completed. The mixtures were centrifuged at 16,000 x g for 10 min at 4°C and the supernatant was collected for subsequent ^1^H NMR spectroscopic analysis. A total of 400 μL fecal water extract or urine was transferred into a clean 1.5 mL microtubes and mixed with 250 μL of 0.2 M sodium phosphate buffer (100% D_2_O, 0.01% TSP (3-(trimethylsilyl)propionic-2,2,3,3-d_4_ acid sodium salt) as a chemical shift reference, and 3 mM sodium azide (NaN_3_) as a bacteriostatic reagent, pH 7.4). The mixture was vortexed and centrifuged at 16,000 x g for 10 min at 4°C and 600 μL of supernatant was transferred into NMR tube with an outer diameter of 5 mm.

^1^H NMR spectra of all fecal samples were acquired using a 600 MHz spectrometer (Bruker Avance III, Bruker Biospin, Germany) with 5 mm broadband inverse configuration probe with a z axis magnetic field-gradient capability operating at 600.13 MHz for proton. D_2_O in the sodium phosphate buffer was used to lock the magnetic field. A standard 1-dimensional (1-D) NMR pulse [recycle delay (RD)-90°-t_1_-90°-t_m_-90°-acquire free induction decay (FID)] was employed at 300 K for the acquisition of fecal water spectra. A total of 128 scans were recorded into 64 k data points with a spectral width of 20 ppm.

Urine samples were prepared by combining 400 μL of urine with 250 μL of 0.2 M sodium phosphate buffer (100% D_2_O, 0.01% TSP, and 3 mM sodium azide (NaN_3_), pH 7.4). The mixture was vortexed and centrifuged at 16,000 x g for 10 min at 4°C and 600 μL of supernatant was transferred into NMR tube with an outer diameter of 5 mm pending for NMR analysis. ^1^H NMR spectra of all urinary samples were acquired using a 600 MHz spectrometer (Bruker Avance III, Bruker Biospin, Germany) with 5 mm broadband inverse configuration probe with a z axis magnetic field-gradient capability operating at 600.13 MHz for proton. D_2_O solvent in the sample buffer was used to lock the magnetic field. A standard 1-dimensional (1-D) NMR pulse [recycle delay (RD)-90°-t_1_-90°-t_m_-90°-acquire free induction decay (FID)] was employed at 300 K for the acquisition of urine spectra. A total of 128 scans were recorded into 64 k data points with a spectral width of 20 ppm.

Plasma samples were first vortexed before 400 μL was combined with 250 μL of saline solution (0.9% NaCl w/v, 20% D_2_O). The mixture was vortexed and centrifuged at 16,000 x g for 10 min at 4°C before 600 μL of the supernatant was transferred to a 5 mm outer diameter NMR tube. A Carr−Purcell−Meiboom−Gill (CPMG) pulse sequence [RD−90°−(ꞇ − 180°−ꞇ)_n_− acquisition] was applied to plasma samples at 310 K (2ꞇn = 76.8 ms) to improve the visualization of signals generated from low molecular weight metabolites. A total of 32 scans were recorded into 72 k data points with a spectral width of 20 ppm.

## NMR spectral data analysis

^1^H NMR spectra obtained from the rat samples were automatically pre-processed in TopSpin 3.1 software which included phasing, baseline correction and referencing to TSP peak in urinary and fecal spectra at δ^1^H 0.00 or the anomeric proton from α-glucose at δ^1^H 5.223 in plasma spectra. The high-resolution integration of peaks in the spectra creates huge data matrices which require powerful software; MATLAB (MathWorks) has been employed in this study. The NMR spectra were subsequently transferred into MATLAB software and digitized into 20 k data points with a resolution of 0.0005 ppm. The water peak was excised from urinary (δ^1^H 4.72 and 4.90), fecal (δ^1^H 4.50 and 5.10) and plasma (δ^1^H 4.50 and 5.10) spectra to minimize the effect of the remaining baseline distortion caused by imperfect water suppression. In addition, regions between δ^1^H 5.45 and 6.15 containing urea signals were removed from urinary spectra. The pre-processed spectral data of urine and feces were aligned to adjust for shifts in peak position^58^ due to small pH differences between samples and normalized using median fold normalization.^59^ Citrate (δ^1^H 2.48 and 2.70) was removed from plasma spectra prior to the peak alignment and the plasma spectra were not normalized since blood composition is maintained under homeostatic control.

The resulting NMR spectra were imported into SIMCA 14.0 (Umetrics) to conduct principal component analysis (PCA) with a unit variance (UV) scaling method, followed by an orthogonal signal correction-projection to latent structures-discriminant analysis (O-PLS-DA) in MATLAB (R2014a) environment using in-house developed scripts. MATLAB was used to generate O-PLS-DA coefficient plots, with a color visualization of correlation values (r^2^) of each variable. Red color indicates higher correlation whilst blue indicates lower correlation of the variables with the classification. The fitness and predictability of the models obtained from OPLS-DA were determined by the R^2^ and Q^2^ values, respectively. The O-PLS-DA models in the current study were established based on one PLS component and one orthogonal component using mean-centered and UV-scaled spectral data sets. Metabolic profiles of urine, feces at all time points and peripheral plasma at the last time point from both lean and obese Zucker rats were correlated with body weight using O-PLS regression analysis. Fasting insulin levels from all groups were statistically correlated with urinary and fecal profiles at the last time point, and the portal vein and peripheral plasma profiles using O-PLS regression analysis. The validation of all O-PLS-DA and O-PLS models involved in this study was assessed using 1,000-times permutation test. To assist metabolite assignment, J-resolved spectroscopy (JRES) was carried out along with the use of in-house and public databases.^60^ Further assignment of metabolites was also accomplished with the use of statistical total correction spectroscopy (STOCSY) on 1-D spectra.^61^

## Bile acid profiling

Urine and plasma samples were thawed at 4°C overnight and centrifuged at 18,000 g for 15 min at 4°C. A total of 100 µL of the supernatant from each sample was transferred into 0.5 mL Eppendorf 96-deepwell plates and 300 µL of ice-cold methanol was added to each well for protein precipitation. All plates were heat-sealed (Thermo Fisher Scientific, Hertfordshire UK), vortexed for 30 min at 4°C using an Eppendorf MixMate at 1400 rpm and incubated for 20 min at −20°C, followed by centrifuging at 3486 g (4500 rpm) for 15 min at 4°C. A total of 200 µL of supernatant was transferred to Eppendorf 350 µL microplates, which were subsequently heat-sealed with thermo foil prior to analysis. Another 20 µL of supernatant from each urine or serum sample was pooled together in glass beakers to form the quality control (QC) samples (total volume = 1420 µL for each type of biofluid). A total of 4260 µL of ice-cold methanol was added to the glass beaker for protein precipitation according to the aforementioned incubation steps. The supernatant from the QC sample was transferred into multiple wells in the Eppendorf 350 µL microplates.

Bile acid profiling was carried out using ACQUITY UltraPerformance Liquid Chromatography (UPLC) coupled with a Xevo G2-S Q-ToF mass spectrometer (Waters Ltd.). The injection volume of all samples was 10 μL. To minimize injector carry-over, 3 wash cycles of weak (H_2_O:2-propanol, 9:1, v:v) and strong (2-propanol) solvent preparations were performed simultaneously with sample analysis. ACQUITY BEH C8 column (1.7 μm, 100 mm × 2.1 mm) was used at an operating temperature of 60°C. The mobile phase A consisted of acetonitrile and water (UPLC grade) (1:10, v:v) with 1 mM ammonium acetate and pH was adjusted to 4.15 with acetic acid. Mobile phase B consisted of acetonitrile and 2-propanol (1:1, v:v). The liquid chromatography condition was adopted from a previous publication by Sarafian *et al.^62^* In brief, the gradient was started with 90% A at an initial flow rate of 0.6 mL/min for 0.1 min, followed by a linear reduction of A to 65% from 0.1 to 9.25 min and another further reduction to 15% from 9.25 to 11.5 min. Between 11.5 and 11.8 min, the flow rate was increased to 0.65 mL/min and the solvent A was reduced from 15% to 0%. Between 11.8 and 12.4 min, 100% B was used to wash off the lipidic matrix at a flow rate of 0.8–1 mL/min. Between 12.45 and 15 min, the column was conditioned to the initial condition and the flow rate was reduced to 0.6 mL/min. The MS system was equipped with an electrospray ionization source operating in negative ion mode (ESI−). Mass spectrometry parameters were as follows: capillary voltage was set at 1.5 kV, cone voltage at 60 V, source temperature at 150°C, desolvation temperature at 600°C, desolvation gas flow at 1000 L/h, and cone gas flow rate at 150 L/h. Seven injections of solvent blanks and 10 injections of the QC sample were carried out before the analysis of the samples. The QC sample was injected once every 11 sample injections. Thirteen mixtures of bile acid standards were injected at the end of the sample run for identification. Automatic data dependent acquisition, MSE and dynamic range enhancement MS methods were applied to the QC sample for bile acid identification.

## Bile acid profile analysis

The raw data were converted to NetCDF format using Databridge built in MassLynx V4.1 (Waters, Inc) and the data were extracted using XCMS package in R software. The relative levels of the identified bile acids, reflected by relative peak intensities, were analyzed using multiple Student’s t-test.

## 16S rRNA gene-based sequencing analysis

Bacterial genomic DNA was isolated from gastrointestinal content and fecal samples using a PowerFecal® DNA isolation kit (MOBIO Laboratories, Inc., USA). A total weight of approximately 0.25 g was transferred to the prefilled-dry bead tube followed by the addition of 750 μL of bead solution and 60 μL of solution C1 containing sodium dodecyl sulfate (SDS) prior to homogenization and cell lysis. The mixture was heated at 65°C for 10 minutes and subsequently bead-beaten at 5500 Hz for 2 cycles x 20 seconds. The mixture was centrifuged at 13,000 x g for 1 minute and approximately 400 μL of supernatant was transferred to a clean 2 ml collection tube.

DNA sequencing was undertaken by Research and Testing Laboratory (Austin, Texas, USA) using the Illumina MiSeq. In brief, to determine the diversity and structure of the bacterial communities in different samples, the protocol as previously described by Caporaso *et al*.^63^ was used. PCR amplifications were performed with the primers 515 F (5-GTGCCAGCMGCCGCGGTAA-3ʹ) and 806 R (5-GGACTACHVGGGTWTCTAAT-3ʹ), which contains a 6-bp error-correcting barcode unique to each sample, for the V4 region of the 16S rRNA gene. The PCR products from different samples were quantified using a Qubit 2.0 fluorometer (Invitrogen, Carlsbad, CA) and mixed accordingly to achieve the equal concentration in the final mixture, which was used to construct PCR amplicon libraries. To minimize the impact of potential early round PCR errors, 20 independent PCR products for each sample were combined to construct PCR amplicon libraries. Sequencing was performed on an Illumina MiSeq platform.

The 16S rRNA gene sequences were analyzed using the bioinformatics software package Mothur and the MiSeq SOP Pipeline. 16S rRNA gene sequence reads were quality checked and normalized to the lowest number of reads in Mothur. To maintain normalization and minimize artifacts, singletons and any Operational Taxonomic Units (OTUs), which were not found at >10 OTUs in any one sample, were collated as OTU singletons and OTU_rare phylotypes. Using the Vegan package of the R statistical package, analysis was performed on the data sets contained within the files generated by Mothur (all OTUs were defined using a cutoff value of 97%). The Unifrac-weighted distance matrix was analyzed in R using nonmetric multidimensional scaling ordination and the shared OTU file was used to determine the number of times that an OTU was observed in multiple samples, and was used for multivariate analysis in R. Operational Taxonomic Unit taxonomies (from phylum to genus) were determined using the RDP MultiClassifier script to generate the RDP taxonomy. Alpha and beta indices were calculated from these datasets with Mothur and R using the Vegan package. The OTU data were subsequently analyzed using STAMP.^64^

## Supplementary Material

Supplemental Material

## Data Availability

The raw 16S rRNA sequencing data is available at the European Nucleotide Archive with the accession number: PRJEB41988. https://www.ebi.ac.uk/ena/browser/view/PRJEB41988
