# Supplementary material for: Roux-en-Y gastric bypass surgery in Zucker rats induces bacterial and systemic metabolic changes independent of caloric restriction-induced weight loss
Source: Gut Microbes. 2021 Feb 4;13(1):1875108. doi: 10.1080/19490976.2021.1875108 (PMC7872092; doi:10.1080/19490976.2021.1875108)
Supplement: Supplemental Material [file KGMI_A_1875108_SM4124.docx]

**Supplementary information**

**Roux-en-Y gastric bypass surgery in Zucker rats induces bacterial and systemic metabolic changes independent of caloric restriction-induced weight loss**

Florian Seyfried^a,‡^, Jutarop Phetcharaburanin^b,c,‡^, Maria Glymenaki^b,‡^, Arno Nordbeck^a^, Mohammed Hankir^a^, Jeremy K Nicholson^d^, Elaine Holmes^b,d^, Julian R. Marchesi^b,e^, & Jia V. Li^b,*^

^a^Department of General, Visceral, Transplant, Vascular, and Pediatric Surgery, University Hospital Wuerzburg, Wuerzburg, Germany; ^b^Department of Metabolism, Digestion and Reproduction, Faculty of Medicine, Imperial College London, London, United Kingdom; ^c^Department of Biochemistry, Faculty of Medicine, Khon Kaen University, Khon Kaen, Thailand; ^d^Institute of Health Futures, Murdoch University, Perth, Western Australia, Australia; ^e^School of Biosciences, Cardiff University, Cardiff, United Kingdom.

^‡^These authors contributed equally to this work

*Corresponding author. Tel: +44 20 7594 3230; E-mail: [jia.li@imperial.ac.uk](mailto:jia.li@imperial.ac.uk)

**Figure S1 Comparison of microbial diversity metrics between RYGB (blue), Sham-obese (Red) and Sham-BWM (green) at pre-operation, and 1 and 4 weeks post-operation.**

Shannon diversity index of Sham-BWM is significantly higher than RYGB and Sham-obese group at week 4 post-operation with a p value of 0.0087 (**) and 0.0159 (*) based on Wilcoxon test. The middle line in the box represents the median value. The lower and upper hinges represent 25% and 75% percentiles and the whiskers represent 1.5 x inter-quartile range (IQR) from each hinge.

**Figure S2 Impact of RYGB and caloric restriction on fecal bacterial composition**

PCA plots of fecal family-level taxonomic profiles from RYGB, sham-obese and sham-BWM groups at pre-surgery (Pre), and 1- (1wk) and 4-weeks (4wk) post-surgery (A). At each time point, bar plots were generated based on significantly different bacterial families between RYGB and sham-BWM or sham-obese using White’s non-parametric t-test, two-sided and multiple test corrected using Benjamini-Hochberg FDR (B). No significant differences were observed between sham-obese and sham-BWM at any time points. Otux4 represents the sum of the singletons.

Figure S3. Dynamic shifts induced by RYGB or caloric restriction in fecal (A) and urinary (B) metabolic profiles of Zucker obese rats

PCA scores plots of the fecal (A) and urinary (B) metabolic profiles of RYGB (blue), Sham-BWM (green) and sham-obese (red) Zucker rats at pre-operation (square), 1- (triangle), 2- (diamond) and 4-week (dot) post-operation. Percentage on each PC represents variation explained by each principal component.

Figure S4. Impact of the RYGB surgery on fecal metabolic profiles of Zucker obese rats

PCA scores plots (left panel) and OPLS-DA cross-validated scores plots (right panel) of the fecal metabolic profiles between RYGB (blue) and sham-obese (red) Zucker rats at pre-operation (square), 1- (triangle), 2- (diamond) and 4-week (dot) post-operation. For PCA scores plots, percentage on each PC represents variation explained by each principal component and Q^2^ represents overall cross-validated R^2^X for two components. For OPLS-DA cross validated scores plots, p values were derived from CV-ANOVA. The Q^2^Y, R^2^X and p values for the models are summarized in Table S1.

Figure S5. Body weight loss-independent impact of the RYGB surgery on fecal metabolic profiles of Zucker obese rats

PCA scores plots (left panel) and OPLS-DA cross-validated scores plots (right panel) of the fecal metabolic profiles between RYGB (blue) and sham-BWM (green) Zucker rats at pre-operation (square), 1- (triangle), 2- (diamond) and 4-week (dot) post-operation. For PCA scores plots, percentage on each PC represents variation explained by each principal component and Q^2^ represents overall cross-validated R^2^X for two components. For OPLS-DA cross validated scores plots, p values were derived from CV-ANOVA. The Q^2^Y, R^2^X and p values for the models are summarized in Table S1.

Figure S6. Impact of the RYGB surgery on urinary metabolic profiles of Zucker obese rats

PCA scores plots (left panel) and OPLS-DA cross-validated scores plots (right panel) of the urinary metabolic profiles between RYGB (blue) and sham-obese (red) Zucker rats at pre-operation (square), 1- (triangle), 2- (diamond) and 4-week (dot) post-operation. For PCA scores plots, percentage on each PC represents variation explained by each principal component and Q^2^ represents overall cross-validated R^2^X for two components. For OPLS-DA cross validated scores plots, p values were derived from CV-ANOVA. The Q^2^Y, R^2^X and p values for the models are summarized in Table S1.

Figure S7. Body weight loss-independent impact of the RYGB surgery on urinary metabolic profiles of Zucker obese rats

PCA scores plots (left panel) and OPLS-DA cross-validated scores plots (right panel) of the urinary metabolic profiles between RYGB (blue) and sham-BWM (green) Zucker rats at pre-operation (square), 1- (triangle), 2- (diamond) and 4-week (dot) post-operation. For PCA scores plots, percentage on each PC represents variation explained by each principal component and Q^2^ represents overall cross-validated R^2^X for two components. For OPLS-DA cross validated scores plots, p values were derived from CV-ANOVA. The Q^2^Y, R^2^X and p values for the models are summarized in Table S1.

Table S1. A summary of the statistical parameters of the O-PLS-DA models for pairwise comparisons based on the ^1^H NMR spectral data. Abbreviations: P_s_, peripheral plasma; and P_p_, portal vein plasma; Pre-op, pre-operation; Post-op, post-operation.

| **Biofluid** | **Groups** | **Time point** | **O-PLS-DA statistical parameters** | | |
| --- | --- | --- | --- | --- | --- |
|  |  |  | **R^2^X** | **Q^2^Y** | **CV-ANOVA**  **p value** |
| **Faeces** | RYGB vs. Sham-obese | Pre-op | 52.8% | <0 | >0.05 |
|  | RYGB vs. Sham-obese | 1-week post-op | 57.5% | 0.79 | 0.001 |
|  | RYGB vs. Sham-obese | 2-week post-op | 55.1% | 0.74 | 0.003 |
|  | RYGB vs. Sham-obese | 4-week post-op | 59.1% | 0.84 | <0.001 |
|  | RYGB vs. Sham-BWM | Pre-op | 51.3% | <0 | >0.05 |
|  | RYGB vs. Sham-BWM | 1-week post-op | 27.8% | 0.85 | <0.001 |
|  | RYGB vs. Sham-BWM | 2-week post-op | 55.2% | 0.78 | 0.001 |
|  | RYGB vs. Sham-BWM | 4-week post-op | 59.2% | 0.82 | <0.001 |
|  | Sham-BWM vs. Sham-obese | Pre-op | 58% | <0 | >0.05 |
|  | Sham-BWM vs. Sham-obese | 1-week post-op | 37.6% | 0.14 | >0.05 |
|  | Sham-BWM vs. Sham-obese | 2-week post-op | 45.3% | 0.28 | >0.05 |
|  | Sham-BWM vs. Sham-obese | 4-week post-op | 44.7% | 0.35 | >0.05 |
| **Urine** | RYGB vs. Sham-obese | Pre-op | 26.9% | <0 | >0.05 |
|  | RYGB vs. Sham-obese | 1-week post-op | 36.2% | 0.84 | <0.001 |
|  | RYGB vs. Sham-obese | 2-week post-op | 40.5% | 0.87 | <0.001 |
|  | RYGB vs. Sham-obese | 4-week post-op | 42.5% | 0.81 | <0.001 |
|  | RYGB vs. Sham-BWM | Pre-op | 19.5% | <0 | >0.05 |
|  | RYGB vs. Sham-BWM | 1-week post-op | 32.1% | 0.78 | 0.001 |
|  | RYGB vs. Sham-BWM | 2-week post-op | 35.6% | 0.80 | <0.001 |
|  | RYGB vs. Sham-BWM | 4-week post-op | 39.1% | 0.75 | 0.002 |
|  | Sham-BWM vs. Sham-obese | Pre-op | 32.3% | <0 | >0.05 |
|  | Sham-BWM vs. Sham-obese | 1-week post-op | 32.8% | 0.54 | >0.05 |
|  | Sham-BWM vs. Sham-obese | 2-week post-op | 45.6% | 0.45 | >0.05 |
|  | Sham-BWM vs. Sham-obese | 4-week post-op | 53.3% | 0.66 | >0.05 |
| **Plasma** | RYGB (P_s_) vs. RYGB (P_p_) | 4-week post-op | 28.4%% | 0.44 | 0.03 |
|  | Sham-BWM (P_s_) vs. Sham-BWM (P_p_) | 4-week post-op | 33.4 % | 0.51 | >0.05 |
|  | Sham-Obese (P_s_) vs. Sham-Obese (P_p_) | 4-week post-op | 38.2% | 0.67 | >0.05 |
|  | Sham-BWM (P_s_) vs. Sham-obese (P_s_) | 4-week post-op | 35.3% | 0.43 | >0.05 |
|  | RYGB (P_s_) vs. Sham-obese (P_s_) | 4-week post-op | 33.5% | 0.64 | 0.02 |
|  | RYGB (P_s_) vs. Sham-BWM (P_s_) | 4-week post-op | 32.3% | 0.77 | 0.003 |
|  | Sham-BWM (P_p_) vs. Sham-obese (P_p_) | 4-week post-op | 35.1% | 0.3 | >0.05 |
|  | RYGB (P_p_) vs. Sham-obese (P_p_) | 4-week post-op | 30.9% | 0.65 | 0.01 |
|  | RYGB (P_p_) vs. Sham-BWM (P_p_) | 4-week post-op | 27% | 0.8 | <0.001 |

Table S2. A summary of the significantly changed bile acids observed in peripheral plasma (P_s_) or portal vein plasma (P_p_) profiles between RYGB and sham-obese groups, and between RYGB and sham-BWM groups at 4-week post-surgery. ↑ indicates higher relative concentrations of bile acids in sham groups or in portal vein plasma. Blank cells indicate non-significant changes observed.

| **Bile acids (1^st^, primary bile acids; 2^nd^, secondary bile acids)** | **m/z_retention time (min)** | **P_s_** | | **P_p_** | | | **P_s_ vs. P_p_** | | |
| --- | --- | --- | --- | --- | --- | --- | --- | --- | --- |
|  |  | **RYGB vs. Sham-obese** | **RYGB vs. Sham-BWM** | **RYGB vs. Sham-obese** | **RYGB vs. Sham-BWM** | **Sham-BWM** | | **RYGB** | **Sham-ob** |
| 12 Dehydrocholic acid | 407.2713_6.14 |  |  |  |  |  | |  | ↑ |
| 3 Dehydrocholic acid | 405.2635_7.21 |  |  |  |  |  | |  |  |
| 3α-Hydroxy-12 ketolithocholic acid | 389.2688_8.86 |  |  |  |  | ↑ | | ↑ |  |
| 3α-Hydroxy-7 ketolithocholic acid | 389.2688_8.55 |  |  | ↑ |  |  | |  |  |
| 5α-Cholanic acid-3α-ol-6-one | 389.2681_8.01 |  |  |  |  |  | |  |  |
| 5β-Cholanic acid-3α, 6α-diol-7-one | 405.2636_6.63 |  |  |  |  |  | |  |  |
| 5β-Cholenic Acid-7α-ol-3-one | 389.269_9.89 |  |  |  |  | ↑ | |  |  |
| Chenodeoxycholic acid (1°) | 391.2848_10.23 | ↑ |  | ↑ |  |  | |  | ↑ |
| Cholic acid (1°) | 407.2803_8.33 |  |  | ↑ |  | ↑ | |  |  |
| Deoxycholic acid (2°) | 391.2847_10.35 |  |  |  |  | ↑ | |  | ↑ |
| Glycochenodeoxycholic acid (1°) | 448.3063_7.61 |  |  |  |  |  | |  |  |
| Glycodeoxycholic acid (2°) | 448.3069_8.08 |  |  |  |  |  | |  |  |
| Glycohyodeoxycholic acid (2°) | 448.3061_5.55 |  |  |  |  |  | |  |  |
| Glycoursodeoxycholic acid (2°) | 448.3063_5.18 |  |  |  |  |  | |  |  |
| Hyocholic acid (1°) | 407.2787_7.78 |  |  |  |  |  | |  |  |
| Hyodeoxycholic acid (2°) | 391.2845_8.61 | ↑ |  | ↑ |  |  | |  |  |
| Isomer of 3a-hydroxy-6,7-Diketocholanic acid sulfate | 483.3184_10.87 |  |  |  |  |  | |  |  |
| Isomer of glycocholic acid (1°) | 464.2933_11.1 |  |  |  |  |  | |  |  |
| Isomer of glyco-lithocholic acid sulfate (2°) | 512.2698_3.23 |  |  |  |  |  | |  |  |
| Murocholic acid (1°) | 391.2842_7.29 |  |  | ↑ |  |  | |  |  |
| Tauro ω-muricholic acid* (1°) | 514.2717_3.34 |  |  |  |  | ↑ | |  | ↑ |
| Taurochenodeoxycholic acid (1°) | 498.2899_6.95 | ↑ |  |  | ↑ | ↑ | | ↑ |  |
| Taurocholic acid (1°) | 514.2858_5.15 |  |  |  |  |  | |  |  |
| Taurodeoxycholic acid (2°) | 498.29_7.39 |  |  |  |  |  | |  |  |
| Taurohyodeoxycholic acid (2°) | 499.2928_4.94 | ↑ |  | ↑ | ↑ |  | |  |  |
| Tauro-ursocholanic acid | 482.2947_9.08 |  |  |  |  | ↑ | |  | ↑ |
| Tauro-ursodeoxycholic acid (2°) | 498.2896_4.63 |  |  |  |  | ↑ | |  | ↑ |
| Tauro-α muricholic acid (1°) | 514.2846_3.19 |  |  |  |  | ↑ | |  |  |
| Tauro-β muricholic acid (1°) | 514.2114_3.18 |  |  |  |  | ↑ | | ↑ |  |
| Ursodeoxycholic acid (2°) | 391.2842_8.08 |  |  |  |  |  | |  |  |
| α-muricholic acid (1°) | 407.2795_6.57 |  |  | ↑ |  |  | |  |  |
| β-muricholic acid (1°) | 407.2793_6.67 |  |  |  |  |  | |  | ↑ |
